# Confronting Views of Companies and Authorities on Food Safety Issues—A Cross-Country Survey

**DOI:** 10.3390/foods13050773

**Published:** 2024-03-01

**Authors:** Ilija Djekic, Garegin Hambardzumyan, Aleksandra Nikolić, Alen Mujčinović, Dimitar Nakov, Aleksandra Silovska Nikolova, Anastasia A. Semenova, Oksana A. Kuznetsova, Fatih Oz, Emel Oz, Nino Terjung, Heinz Volker, Igor Tomasevic

**Affiliations:** 1 Faculty of Agriculture, University of Belgrade, 11080 Belgrade, Serbia; 2 Veterinary Sanitary Examination, Food Safety and Hygiene Department, Armenian National Agrarian University, Yerevan 0009, Armenia; garegin77@gmail.com; 3 Faculty of Agriculture and Food Sciences, University of Sarajevo, 71000 Sarajevo, Bosnia and Herzegovina; a.nikolic@ppf.unsa.ba (A.N.); a.mujcinovic@ppf.unsa.ba (A.M.); 4 Faculty of Agriculture, “Goce Delčev” University in Štip, 2000 Shtip, North Macedonia; dimitar.nakov@ugd.edu.mk; 5 Faculty of Agricultural Sciences and Food, SS Cyril and Methodius University in Skopje, 1000 Shtip, North Macedonia; silovska@fznh.ukim.edu.mk; 6 V. M. Gorbatov Federal Research Center for Food Systems, Moscow 107023, Russia; a.semenova@fncps.ru (A.A.S.); o.kuznecova@fncps.ru (O.A.K.); 7 Faculty of Agriculture, Ataturk University, Erzurum 25030, Türkiye; fatihoz@atauni.edu.tr (F.O.); emel.oz@atauni.edu.tr (E.O.); 8 DIL German Institute of Food Technology, 49610 Quakenbrück, Germany; n.terjung@dil-ev.de (N.T.); v.heinz@dil-ev.de (H.V.)

**Keywords:** food safety, food value chain, food safety integrity, food safety knowledge

## Abstract

This study investigated food safety issues as perceived by food companies and food safety authorities in six countries in Europe and Central Asia. A total of 66 companies and 16 authorities participated in the survey. The results provide important insights related to what the main food safety priorities are, how they are addressed in the countries that participated in the survey, and what the role of the main stakeholders is in the food value chain. Almost 50% of food companies identified ‘food fraud’ as the most influential food safety attribute. One-third of food safety authorities recognized ‘food safety management system’ as the most influential food safety attribute. Principal component analysis separated food safety statements into two dimensions named ‘food safety hazards and risks’ and ‘food safety system’. Although there are slight differences in food safety statements between the two stakeholders, i.e., food companies and food safety authorities, it is the country of origin that plays a more important role in understanding their views. Food companies will need to implement a systemic approach and transform the entire food value chain continuum while considering new food safety challenges. It is expected that food safety authorities will have to play a more proactive role in the future.

## 1. Introduction

Eating safe food that is suitable for consumption is a prominent human right [1], built upon four key elements of the right to food: availability, accessibility, adequacy, and sustainability [2]. Food hygiene is the first pillar in developing and maintaining consumers’ trust in food safety [3]. Widening the perspective of food safety, we can recognize complex push and pull drivers that enforce its development. Food safety legislation introduces new, stricter legal requirements to provide a high level of health protection to consumers, to only allow safe food on the market, and to take care of the environment’s health, namely plant life and animal welfare [4]. In parallel, food science recognizes different elements that make up the concept of food safety throughout the food supply chain continuum, such as the prevention of deliberate food adulteration and counterfeiting threats [5], the development of different food safety culture models [6], and the introduction of the concept of food integrity [7].

In 2016, the Food and Agriculture Organization (FAO) performed a survey of all Codex Alimentarius Commission members of the region of Europe, revealing critical and/or emerging food safety and quality issues. Based on responses from 21 different countries and one coordinated response from the European Union (EU), the following issues were identified [8]: (i) food fraud and food adulteration; (ii) new technologies and climate change; (iii) globalization of trade, contaminants (including mycotoxins), and foodborne pathogens; (iv) antimicrobial resistance; (v) new distribution channels; (vi) challenges in food safety management along the food chain; (vii) food additives.

The most frequently notified types of food safety hazards within the European Rapid Alert System for Food and Feed (RASFF) database in the period 2010–2016 comprise microbial (pathogenic microorganisms), chemical (mycotoxins, pesticide residues, heavy metals, residues of veterinary medicinal products) and physical (foreign bodies) hazards [9]. During 2017–2020, significant notifications on pathogenic microorganisms were observed, with *Salmonella* being the most frequently reported pathogen, followed by *Listeria monocytogenes*, Shiga toxin-producing *Escherichia coli*, and norovirus. Pesticide residues, together with the presence of heavy metals, were also a trend observed in the database. Furthermore, in 2018, allergens (milk, gluten, and nuts) and foreign bodies (plastic, metal, and glass) started to pop up, increasing yearly.

Regarding official monitoring in non-EU member countries, the information shows different patterns. Mycotoxins were the most frequently reported food product hazard, such as aflatoxins from Turkish dried figs and ochratoxin A in fruits and vegetables [9]. In Türkiye, data about foodborne diseases are scarce due to the low level of reporting by official authorities and the difficulty of accessing official statistics on food safety incidents. The Turkish Health Minister recently noted in an interview that between 2015 and 2020, over 18 million people were hospitalized due to foodborne diseases, with over 1700 people dead [10]. The places that were most frequently reported as centers of foodborne poisoning outbreaks were school and university canteens [11]. Besides microbial outbreaks, pesticides are another major food safety issue, since the same Turkish Ministry reported health problems for more than 10,000 people in the period between 2013 and 2019, with almost 50 fatalities [12].

In 2018–2020, around two percent of examined food samples in North Macedonia did not comply with local food safety regulations. In addition, chemical hazards compromised samples in relation to increased levels of food additives and confirmed contamination with heavy metals. Microbial pathogens that served as outbreak vectors were *Salmonella*, *Campylobacter*, *Escherichia coli* beta glucuronidase, and *Listeria monocytogenes*, alongside mycotoxins from the group of aflatoxins B1, B2, G1, and G2 [13,14]. As a result, the Institute for Public Health organized food safety and environmental protection training for over 400 trainees in 2021 [15].

In Serbia, official data about food safety issues and foodborne disease cases and outbreaks are part of an annual report of the Institute of Public Health regarding contagious diseases. According to these reports, salmonellosis incidents rates are high, and campylobacteriosis is the second most frequent enteropathy, with moderate records of trichinellosis outbreaks and rare notices of shigellosis and botulism [16,17]. Furthermore, when it comes to other food safety hazards, the Directorate for Veterinary Inspection reported veterinary medicinal product residues and other substances in live animals and foods of animal origin [18].

In the Russian Federation, pathogenic microorganisms are recognized as the dominant vector for food safety issues, highlighting salmonellosis as the most important infective agent of all pathogenic microorganisms in foodborne disease outbreaks, especially their morbidity rates. According to official results of epidemiological investigations of salmonellosis outbreaks, restaurants and catering businesses were most frequently identified [19,20,21,22]. In parallel, the incidence of campylobacteriosis in the last few years has grown. At the same time, the number of clinically diagnosed and laboratory-confirmed cases of listeriosis in the Russian Federation has declined [19,20].

Considering different food safety hazards in non-EU countries underlines the need for investigating priority issues and trends in food safety in Europe and Central Asia. The two main research questions are as follows: (i) what are the main food safety priorities of food companies in the sampled countries? (ii) what are the main food safety priorities of food safety authorities? The working hypothesis is that the two stakeholders have different opinions on food safety priorities. To perform such a study, a survey was performed to interview food safety authorities and value chain actors in the food supply chain of six selected countries.

## 2. Materials and Methods

### 2.1. Survey Characteristics

An online survey was performed using the (Slido^®^) platform in six countries from the Europe and Central Asia region, interviewing two types of respondents: (i) food safety authorities (at institutional/regulatory level); and (ii) medium-sized and/or large food companies in the food value chain. The main criteria for companies were that they were exporters of food, held some recognized food safety management system (FSMS) certificates, and had at least 50 employees (to be categorized as medium-sized companies). This criterion was selected since small food companies lack resources for implementing effective FSMSs [23,24]. The survey was performed during the second half of 2022.

A breakdown of the countries and respondents covered in this survey is depicted in Table 1. The main criteria for inclusion in this paper were that at least ten food companies and at least two food safety authorities from each country had to have been interviewed.

### 2.2. Online Questionnaire

The questionnaire was developed based on a literature review and consisted of four sections. The literature review consisted of two parts—analysis of publications from scientific databases and grey literature. For the bibliometric analysis of publications within the Web of Science and Scopus databases, three keyword phrases were selected: ‘food safety management’, ‘food safety risks’, and ‘food safety issues’. To narrow the review, the following criteria were applied: (a) the selected period of publication was from 2018 until 2022; (b) only articles and review papers were considered; and (c) a text-mining concept using the software tool VOSViewer(version 1.6.19) as an analytic tool was employed. In parallel, a grey literature review covering the six countries that participated in this research was performed using sources from the Internet, such as reports and documents available from the official websites of international organizations and research institutions, such as the World Trade Organization, Organization for Economic Co-operation and Development, World Health Organization, Food and Agriculture Organization, etc.

The Section 1 of the questionnaire captured the demographic characteristics of the respondents (country of origin and whether the interviewee was from a food company or a representative of a food safety authority). The Section 2 consisted of open questions directed to both types of stakeholders in relation to selected challenges/issues: (i) food safety legislation; (ii) food control management/governance with an emphasis on food laboratories and food safety management systems; (iii) combating food safety hazards and risks; and (iv) food safety knowledge.

The Section 3 comprised 24 food safety statements revealed during the literature review, in line with similar previously published research [23,25,26,27,28,29] and international FSMS standards [30,31,32]. The interviewees rated their level of agreement based on a five-point Likert scale from 1 ‘strongly disagree’, through 2 ‘disagree’, 3 ‘no opinion’, and 4 ‘agree’, to 5 ‘strongly agree’ (Table 2).

Food safety priorities were addressed in the Section 4. Based on the literature review, in line with food safety related publications [23,29,33,34], nine categories were selected as most/least influential, as follows: food safety management system (FSMS); food safety knowledge; work of food safety inspection services; new food-related technologies, materials, and packaging; food safety legislation; food fraud; food control laboratories and food analysis; access to food safety and quality information; the role of food science.

When this type of scaling is used (so-called best–worst), each category should be repeated in a subset of choices three to five times [35,36]. In this survey, all categories were available for choice three times, except for FSMS, which was available four times. As a result, seven subsets, each with four categories, were created. Table 3 presents one of the seven subsets.

### 2.3. Statistical Processing of Data

The principal component analysis method (PCA) was used to analyze food safety statements. The reliability of item scales was determined by calculating Cronbach’s α coefficient. To test whether those data are likely factorizable, Bartlett’s test was performed. Eigenvalue was the main criterion in defining the number of PCA components.

As presented, nine food safety categories could be chosen as most influential (best) or least influential (worst). The first step was to count the number of times a certain category was selected. Based on this, it was possible to compute an ‘*S*’ score (Equation (1)) based on Merlino et al. [36] and Djekic et al. [23].

(1) $$S=\frac{B-W}{a\times n}$$

*B*—most frequently chosen; *W*—least frequently chosen; *a*—availability in the series of seven sets (in this survey, the category ‘FSMS’ was available in four subsets, and all other characteristics were available in three subsets); *n*—number of respondents.

To enable more profound conclusions, two additional indices were computed: ‘*B*-*W*%’ (presenting the percentages of categories selected as ‘best’, ‘worst’, and not selected) and ‘preference share’ (showing the likelihood of a category being identified as ‘best’) [35]. The level of statistical significance was set at 0.05.

## 3. Results and Discussion

### 3.1. Literature Review

For the keyword ‘food safety management’, the literature search of articles and review papers for the period 2018–2022 revealed a total of 8570 publications. The top five countries/regions were the USA, with 2075 publications (24.2% of total publications), China, with 1738 publications (20.28%), Italy, with 751 publications (8.76%), the United Kingdom, with 665 publications (7.76%), and Germany, with 591 publications (6.89%). The analysis of the number of publications associated with countries that participated in the survey showed the following: Serbia had 172 publications (2.0% of total publications), Russia had 164 publications (1.91%), and Türkiye had 147 publications (1.71%). The remaining three countries (North Macedonia, Bosnia and Herzegovina, and Armenia) had fewer than 25 publications per country (<0.5%). The top five journals with the most publications were *Food Control*, the *International Journal of Environmental Research and Public Health*, *Foods*, *Ecotoxicology*, *Environmental Safety*, and the *British Food Journal*.

For the keyword ‘food safety risks’, the literature search of articles and review papers for the same period revealed a total of 7047 publications. The top five countries/regions were the USA, with 1652 publications (23.44% of total publications), China, with 1178 publications (16.71%), Italy, with 479 publications (6.79%), the United Kingdom, with 366 publications (5.19%), and Germany with 323 publications (4.58%). As for the countries the participated in this research study, the data are as follows: Türkiye had 97 publications (1.37%), Russia 55 publications (0.78%), Serbia 39 publications (0.55%) and all others (Armenia, North Macedonia, and Bosnia and Herzegovina) had below 20 publications (<0.5% of all publications). The top five journals with the most publications were the *EFSA Journal*, *Food Control*, *Food Additives & Contaminants: Part A*, *Food and Chemical Toxicology*, and *Ecotoxicology and Environmental Safety*.

The final search, for the keyword ‘food safety issues’ in the same period (2018–2022), revealed a total of 2123 publications. The top five countries/regions were the USA, with 440 publications (20.72%), China, with 439 publications (20.67%), Italy, with 173 publications (8.14%), the United Kingdom, with 115 publications (5.41%), and Canada, with 97 publications (4.57%). As for the participating countries, Türkiye had 44 publications (2.07%), followed by Russia with 25 publications (1.17%). All other countries (Serbia, Bosnia and Herzegovina, Armenia and North Macedonia) had below 15 publications (<0.5%). The top five journals with the most publications were *Food Control*, *Foods*, *Trends in Food Science and Technology*, the *EFSA Journal*, and the *International Journal of Environmental Research and Public Health.*

An analysis of the affiliations of the publications’ authors showed that among the top 20 universities, the majority of research comes from US and Chinese universities. In Europe, the research centers with significant numbers of publications were the Universities of Wageningen (The Netherlands), Milan (Italy), Ghent (Belgium), London (UK), Belgrade (Serbia), and the French Institute INRAE. Regarding the selected countries, only the University of Belgrade has a significant number of publications related to food safety, followed by the Russian Academy of Science.

**Figure 1:**
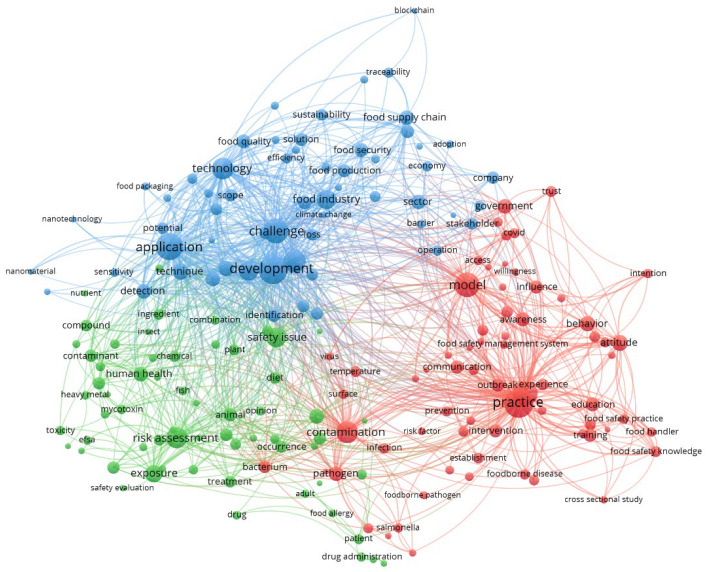
Network visualization of different safety topics based on the three selected keyword phrases (‘food safety management’, ‘food safety risks’, and ‘food safety issues’).

Figure 1 depicts a network visualization of the titles, abstracts, and keywords of the most significant scientific manuscripts published in the last five years globally that included the three selected keyword phrases. It reveals three clusters (presented in different colors). The red cluster, named ‘Practices’, is associated with all kinds of practices that occur in companies, from implementing different food safety systems (HACCP, FSMS) to knowledge of food handlers, intervention/prevention strategies, and various company measures to combat different contaminants, mainly microbial ones. The second cluster, ‘Risks’ (in green), is linked to all types of different hazards and risks that occur in the food sector throughout the food supply chain continuum, such as allergens, various chemical hazards, different legislative measures (local and international, such as those issued by the WHO and EFSA), and exposure assessments. Finally, the blue cluster brings together different aspects of food safety, such as food fraud, various food sustainability dimensions, new technologies (nanomaterials), innovations, and the role of policy makers.

### 3.2. Open Questions

#### 3.2.1. Food Safety Legislation

All respondents (both companies and authorities) confirmed that they believe food safety legislation needs to be more compatible with existing laws (Armenia, North Macedonia) and/or that there is a slow pace of progress towards new legislative initiatives (Russia). When food business operators were asked about possible issues with food legislation in their countries, respondents from Bosnia and Herzegovina noticed that it still lacks full harmonization with EU and other international regulations and standards. Russian food producers perceived it as contradicting in some elements and inconsistent in general, while their North Macedonian colleagues thought that it is too extensive. Serbian food producers found it hard to understand due to the large number of articles that are written in an insufficiently clear manner using terms that are not precisely defined.

All food safety authorities were almost unanimous regarding their lack of resources and competencies and local governments’ inadequate financial support. North Macedonian and Serbian food safety authorities expressed strong concerns due to immense pressure from various food business managers, owners, and politicians, compromising their impartiality. Similar observations were made by authority representatives from Türkiye, who pointed to a lack of authority related to food safety inspectors as opposed to food business operators, fostering potential corrupt practices. Armenian authorities suggested electronic systems to improve efficiency, while their Bosnian and Russian colleagues highlighted bad communication channels between food safety bodies. Serbian food safety authorities expected a shift in monitoring activities and official controls in terms of food safety risk assessment. These countries are generally in different phases of food safety regulatory reforms. Their limited capacities and lack of awareness and knowledge among stakeholders in the food value chain have been confirmed by the FAO [37]. Unlike developed countries that obey their food safety laws, developing and underdeveloped countries struggle with inadequate enforcement methods, bureaucracy, and lack of political will [4].

#### 3.2.2. Food Safety Infrastructure

Most participants agreed that food safety laboratories struggle with inadequate infrastructure, new equipment, competencies, and expertise, and that they need more financial and technical support from national governments. In parallel, an insufficient number of accredited laboratories with validated food safety methods according to ISO 17025 [38] is a problem in most of the surveyed countries. Many food companies stated that the financial costs of laboratory analysis are high, with problems caused by the fact that some laboratory facilities are far away from production sites or even located abroad. In Armenia, food producers expressed doubts about the consistency and accuracy of the results obtained from their local laboratories. Only Serbia reported that there are more laboratories than needed for some types of official control and monitoring activities.

Regarding FSMS, respondents stated that food safety management is needed due to customer demands, exporting markets, and compliance with local food safety rules and regulations. In parallel, most food companies from all surveyed countries replied that their motivation for improving their FSMS is based on the great responsibility of not harming customers and consumers. Furthermore, an interesting approach was put forward by Turkish food authorities, who proposed a penalty–reward system based on the history of safe food production. This supports research by Jaffee et al. [39], who stated that FSMSs tend to be underdeveloped and have limited capacities, except for those in food exporting companies, which strive to develop robust systems supported by the designated ‘competent authorities’. In addition, Turkish scholars [40,41] revealed new food safety dimensions that need to be addressed within implemented FSMSs, from sustainability perspectives to religious criteria in line with halal issues.

#### 3.2.3. Combating Food Safety Hazards and Risks

When it comes to food safety hazards and risks, besides known microbial, chemical, and physical hazards, the respondents raised two important issues: (i) difficulties in maintaining low temperatures during food transportation and retail and (ii) weak traceability systems, as reported in Armenia, Serbia, and Russia. In parallel, the majority emphasized the role of climate change in raising new hazards and risks. Scholars from these countries analyzed mycotoxin and microbial issues associated with climate change. A study from Türkiye confirmed that climatic conditions among regions of Türkiye resulted in the spread of different foodborne mycotoxins, resulting in exceeded limits in various types of food [42]. Serbian scholars identified patterns of climate change and mycotoxins in maize [43]. Climate-related issues were also predicted to result in microbial growth and consequential spoilage of dairy products in Albania, with a scenario of increased outdoor temperature due to climate change [44].

#### 3.2.4. Food Safety Information and Knowledge

Most food safety authorities confirmed that they lack scientific data on food safety due to inadequate information systems and/or official communication channels. Instead, they use the Internet and social networks to share information and knowledge. In Serbia and Türkiye, food safety inspectors exchanged information among themselves using different communication channels, including official governmental websites, email newsletters, targeted trainings, and awareness campaigns. However, when food producers were asked the same question, they almost unanimously reported that established channels of accessing food safety and quality information are inefficient. As a result, they lack information regarding food regulations or changes and amendments to these, while only being notified about them by the authorities post festum. As a result, they are forced to look for information in alternative, non-governmental sources, like privately administered websites or blogs, and/or attend training courses organized by local universities and/or food consulting agencies. To overcome this problem, food producers in Serbia recommended more training activities organized by competent authorities. In Türkiye, they suggested booklets and leaflets on the topic, while in Armenia, they would like to see more public media, like television and newspapers, used for this purpose. The lack of competencies throughout the food supply chain was emphasized as the most important food safety issue, pointing to the need for competent food safety legislators, inspectors, and employees in food value chain actors, food authorities, and food laboratories. Qualified staff are key to effective food safety systems [45]. Therefore, innovative strategies and approaches should enable successful knowledge transfers [33].

### 3.3. Food Safety Statements

Reliability analysis and principal component analysis (PCA) were performed on the set of 24 statements that analyzed the respondents’ level of agreement with certain food safety issues. The reliability of item scales was defined by computing Cronbach’s α coefficient as a measure of internal consistency. Cronbach’s α shows whether a scale is reliable when using multiple questions [46]. The calculated Cronbach’s α was 0.856. Since α ≥ 0.80, it confirms good reliability [47,48].

The overall Kaiser–Meyer–Olkin measure of sampling adequacy was satisfactory (0.739), as when the results of this index are above 0.6, principal component analysis is a useful tool for the given dataset [49]. Bartlett’s test of sphericity was significant (*p* < 0.0005), showing that the data are likely factorizable and confirming an adequate level of correlation between the variables. Based on the PCA, eigenvalue ≥ 1, scree plot, and interpretability criterion, two principal components (PCs) were kept. In addition, a Varimax orthogonal rotation was run to help in interpreting the results. This reduction to two dimensions separated the statements into two distinct directions, and the authors summarized them as ‘food safety hazards and risks’ (PC1) and ‘food safety system’ (PC2). By building on the existing literature that supports food safety, the classification of these two dimensions supports the intertwining of the two pillars.

**Figure 2:**
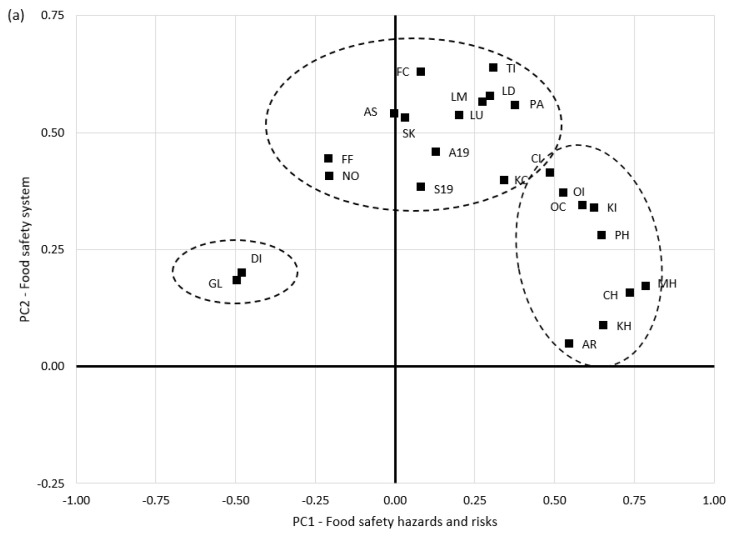

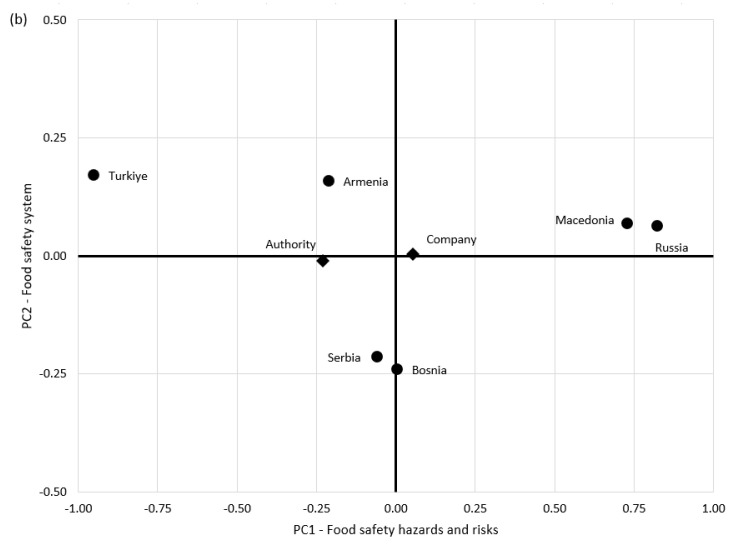
Principal component analysis loadings (**a**) and scores (**b**) plots for the 24 statements deployed by type of respondents and country. Rotation method: Varimix. Statements: (■) Legislation is up to date—LD; Legislation is understandable—LU; Proactive approach—PA; Transparently share information—TI; Knowledge among food inspection services—KI; Knowledge among food handlers—KH; Knowledge among food consumers—KC; Outbreak investigation—OI; Outbreak communication—OC; Assessment/audit services—AS; Scientific knowledge—SK; Laboratory methods—LM; System change in COVID-19—S19; Authority change in COVID-19—A19; Microbial hazards—MH; Chemical hazards—CH; Physical hazards—PH; Antimicrobial resistance—AR; Food fraud—FF; Food safety culture—FC; Continually improving—CI; New distribution channels—DI; Globalization of trade—GL; Novel food technologies—NO. Respondents (♦)—food company and food safety authority; Country (●)—Armenia, Bosnia and Herzegovina, North Macedonia, Russia, Serbia, Türkiye.

A loading plot (Figure 2a) gives a summary of the results. Results are spread across PC1 but show positive loadings for PC2. As such, the results that are grouped show their level of correlation.

The rule of thumb is ‘the farther from the plot origin, the stronger impact the result has’. The component ‘food safety hazards and risks’ (PC1) was loaded (>|0.45|) with microbial, physical, and chemical food safety hazards (AR, MH, CH, PH), food safety knowledge among food handlers and inspection services (KH, KI), and with the statement that that foodborne outbreaks are investigated and communicated adequately (OC, OI). These statements were opposed to new risks arising from globalization and new distribution channels (GT, DI).

On the other hand, the component ‘food safety system’ was positively loaded (>0.50) with the importance of legislation (updated and understandable), the statement that food safety authorities have a proactive approach and transparently share new food safety information, the importance of scientific knowledge related to food safety, the statement that new laboratory methods for combating hazards are being developed, and the statement that food safety culture is a new paradigm expected in food companies (AS, SK, FC, LU, LM, LD, PA, TI). Serbian scholars studied food safety knowledge among students [50] and consumer perception of food allergens [51]. These studies revealed the importance of education in achieving better knowledge regarding food handling practices and food safety, as opposed to a limited knowledge of consumers in understanding ‘may contain traces of an allergen’ on food labels. Two publications on food safety culture in Eastern European countries revealed differences between food companies associated with the EU membership status of the country they operate in, wherein EU companies had a higher level of food safety culture [6,25].

Slightly lower loadings, but still related to this PC (0.35 < loading < 0.50), were found for statements associated with food fraud as an emerging issue to be combated within all food safety systems; novel food technologies, materials, and packaging; changes in food companies and food authorities due to the COVID-19 pandemic; and a lack of knowledge among consumers (FF, NO, S19, A19, KC). The FAO and the WHO [52] reported that new food safety issues on the rise are related to food fraud and the globalization of food chains. Smigic et al. [53] analyzed novel foods associated with new food technologies, highlighting the legislative challenges needed when new/modified/different novel food processing technologies are applied. There is a belief (and hope) that traditional (microbiological, chemical, and physical hazards) and emerging (food fraud and climate change) food safety issues will be successfully resolved by technological innovations, breakthroughs, and scientific advances, including nanotechnology, 3D printing of food, automation, remote sensing, the Internet of Things, Big Data, and Artificial Intelligence [54]. Thus, this is still to be confirmed or denied.

A survey on the effects of COVID-19 on food safety systems that covered most countries investigated in this research study revealed that despite the pandemic, staff awareness and hygiene were still the two most important attributes, while health protocols published by the WHO were of limited importance [23]. However, despite the negative consequences of COVID-19 on food safety in terms of unprecedented challenges to food systems worldwide, some authors identified it as an opportunity to change our food system in ways that are less susceptible to disruption [55]. In addition, to increase their food safety knowledge, consumers often use the Internet [56]. Still, this type of information usually does not correspond to current governmental or scientific recommendations. It may be misleading, outdated, incomplete, inaccurate, and, in extreme cases, deceptive and unethical, since food safety statements online are created by non-food-safety professionals [57]. The food safety knowledge of consumers was studied in Türkiye, revealing a need for additional education and the development of media tools that can support behavioral changes to reduce the risks of foodborne illness [58].

The scores plot in Figure 2b summarizes the relationships between the countries and respondents. As results that are close to each other are similar, it is clear that countries (and their food safety contexts) bring more differences compared to the type of respondents (located close to the center, indicating that they share similar views). For example, North Macedonia and Russia are grouped in one cluster, while Serbia and Bosnia and Herzegovina form another. In addition, Armenia and Türkiye have their food safety specificities.

### 3.4. Best–Worst Analysis

A total of 82 respondents (66 food companies and 16 food safety authorities) participated in this survey. Table 4 depicts the subjective priority assigned to the nine food safety categories by food companies, while Table 5 shows the same analysis for food safety authorities.

This methodology can highlight the most influential food safety category as considered by the respondents. The computed ‘S’ score shows the relative power of a category, and scores near values of ‘+1.0/−1.0’ depict increasing/decreasing power, as opposed to scores near ‘0’, implying no power [59]. Therefore, positive scores show that a certain category was selected as ‘best’ more often than as ‘worst’. It is obvious from the figures that there is a different pattern among positive scores between the two types of respondents. Food companies believe the top three most influential food safety categories are food fraud, FSMS, and food safety legislation. In contrast, food safety authorities rank FSMS, food safety legislation, and food safety knowledge as the most influential. When it comes to food providers and retailers, the International Food Policy Research Institute states that the lack of food safety standards across retailer networks in low- and middle-income countries is a critical food safety issue, with food fraud recognized with utmost importance in the entire food chain [60].

Within the subset of choices, ‘food fraud’ was selected as the most important in almost 50% of cases by food companies but only in one-third of cases by food safety authorities. Its share of preference was over 20% (food companies) and around 15% (food safety authorities). ‘FSMS’ shares a similar percentage of being the most important to food companies and food safety authorities (32.95% and 35.94%, respectively). The share of preference was around 20% for both types of respondents. Regarding the least influential factors, both types of respondents ranked food science, access to food safety information, and new technologies the lowest.

## 4. Conclusions

This study provides important insights related to the main food safety priorities, issues, and trends and how they are addressed in the countries that participated in the survey. This study brought together different perspectives of two major stakeholders—food companies and food safety authorities. Differences were mainly associated with the role of food safety authorities, food safety knowledge, combating foodborne outbreaks, and change management during the pandemic. In parallel, food companies perceived food fraud as the most influential food safety attribute, as opposed to food safety authorities pointing to FSMS.

The takeaway message from this research is the need for a more proactive role of food safety authorities, as they should be the key drivers in improving food safety systems in the value chain. In parallel, food companies should implement a systemic approach to transform the entire food value chain continuum, bearing in mind cross-cutting food safety challenges. This study has two limitations: (i) small companies in participating countries were not included in this study; (ii) only six countries in Europe and Central Asia participated in this study.

## Figures and Tables

**Table 1:** Country breakdown of two types of respondents.

Break Down		Companies	Authorities
Country	Armenia	11	3
	Bosnia and Herzegovina	12	3
	North Macedonia	11	2
	Russian Federation	10	2
	Serbia	11	2
	Türkiye	11	4
	Total	66	16

**Table 2:** Food safety statements.

Food Safety Statements	Short	Abbreviations
Food safety legislation is up to date in my country	Legislation is up to date	LD
Food safety legislation is understandable for implementation to all food actors in the supply chain	Legislation is understandable	LU
Food safety authorities have a proactive approach to improving food safety	Proactive approach	PA
Food safety authorities transparently share information on new and emerging food safety risks	Transparently share information	TI
Food safety knowledge among food inspection services is at a high level	Knowledge of food inspection services	KI
Food safety knowledge among food handlers in companies operating within the food supply value chain is adequate	Knowledge among food handlers	KH
Food safety knowledge among food consumers is adequate	Knowledge among food consumers	KC
Foodborne outbreaks in my country are investigated with appropriate corrective action taken	Outbreak investigation	OI
Foodborne outbreaks in my country are communicated to all stakeholders	Outbreak communication	OC
(Private) Assessment/audit services in my country play a role in improving the food safety levels of audited companies	Assessment/audit services	AS
Food safety management systems are based on scientific knowledge	Scientific knowledge	SK
Laboratory methods have improved in terms of use of detecting new food safety hazards	Laboratory methods	LM
Food safety systems in companies in my country have changed due to the COVID-19 pandemic	System change in COVID-19	S19
Work of the food safety authority in my country has changed due to the COVID-19 pandemic	Authority changes in COVID-19	A19
Microbial hazards are effectively controlled in food companies in my country	Microbial hazards	MH
Chemical hazards are effectively controlled in food companies in my country	Chemical hazards	CH
Physical hazards are effectively controlled in food companies in my country	Physical hazards	PH
Antimicrobial resistance in the livestock/aquatic sector is effectively controlled in my country	Antimicrobial resistance	AR
Food fraud is an important food safety issue	Food fraud	FF
Food safety culture has improved in the last 12 months in my country	Food safety culture	FC
Food companies are continually improving their management tools in mitigating food safety risks	Continually improving	CI
Do new distribution channels, including e-commerce in my country, bring new levels of food safety risks?	New distribution channels	DI
Does globalization of trade bring new food safety risks?	Globalization of trade	GL
Novel food processing technologies overall improve food safety	Novel food technologies	NO

**Table 3:** Model of a food safety issue subset. All respondents were asked to indicate which of the four presented issues they considered most important (best) and least important (worst).

Most Important	Food Safety Issue	Least Important
**☐**	Food safety knowledge	**☐**
**☐**	Work of food inspection services	**☐**
**☐**	Food safety management system	**☐**
**☐**	Novel technologies/materials/packaging	**☐**

**Table 4:** Subjective priority of food safety issues rated by food companies: best–worst scaling report—frequency counts and standardized average scores (N = 66).

Attributes	Distribution [%]			Share of Preference [%]	
	Most Important	Least Important	Not Chosen		
Food fraud	49.49%	16.16%	34.34%	21.21%	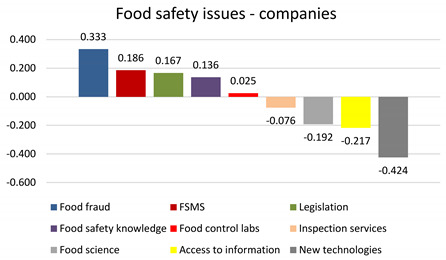
FSMS	32.95%	14.39%	52.65%	18.83%	
Legislation	28.28%	11.62%	60.10%	12.12%	
Food safety knowledge	30.30%	16.67%	53.03%	12.99%	
Food control labs	17.68%	15.15%	67.17%	7.58%	
Inspection services	22.73%	30.30%	46.97%	9.74%	
Food science	12.12%	31.31%	56.57%	5.19%	
Access to information	18.18%	39.90%	41.92%	7.79%	
New technologies	10.61%	53.03%	36.36%	4.55%	

**Table 5:** Subjective priority of food safety issues rated by food authorities: best–worst scaling report—frequency counts and standardized average scores (N = 16).

Attributes	Distribution [%]			Share of Preference [%]	
	Most Important	Least Important	Not Chosen		
FSMS	35.94%	7.81%	56.25%	20.54%	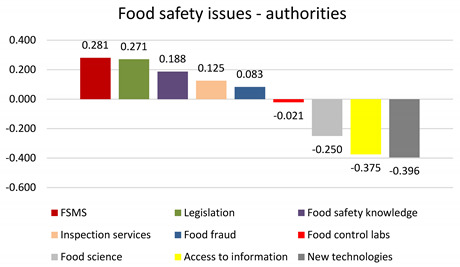
Legislation	41.67%	14.58%	43.75%	17.86%	
Food safety knowledge	43.75%	25.00%	31.25%	18.75%	
Inspection services	22.92%	10.42%	66.67%	9.82%	
Food fraud	33.33%	25.00%	41.67%	14.29%	
Food control labs	16.67%	18.75%	64.58%	7.14%	
Food science	6.25%	31.25%	62.50%	2.68%	
Access to information	18.75%	56.25%	25.00%	8.04%	
New technologies	2.08%	41.67%	56.25%	0.89%	

## Data Availability

The original contributions presented in the study are included in the article, further inquiries can be directed to the corresponding authors.
